# Bevacizumab, an anti-vascular endothelial growth factor antibody, inhibits osteoarthritis

**DOI:** 10.1186/s13075-014-0427-y

**Published:** 2014-09-18

**Authors:** Toshihiro Nagai, Masato Sato, Miyuki Kobayashi, Munetaka Yokoyama, Yoshiki Tani, Joji Mochida

**Affiliations:** Department of Orthopaedic Surgery, Surgical Science, Tokai University School of Medicine, 143 Shimokasuya, Isehara, Kanagawa 259-1193 Japan

## Abstract

**Introduction:**

Angiogenesis is an important factor in the development of osteoarthritis (OA). We investigated the efficacy of bevacizumab, an antibody against vascular endothelial growth factor and an inhibitor of angiogenesis, in the treatment of OA using a rabbit model of anterior cruciate ligament transection.

**Methods:**

First, we evaluated the response of gene expression and histology of the normal joint to bevacizumab treatment. Next, in a rabbit model of OA induced by anterior cruciate ligament transection, we used macroscopic and histological evaluations and real-time polymerase chain reaction (PCR) to examine the responses to intravenous (systemic) administration of bevacizumab (OAB IV group). We also investigated the efficacy of intra-articular (local) administration of bevacizumab in OA-induced rabbits (OAB IA group).

**Results:**

Histologically, bevacizumab had no negative effect in normal joints. Bevacizumab did not increase the expression of genes for catabolic factors in the synovium, subchondral bone, or articular cartilage, but it increased the expression of collagen type 2 in the articular cartilage. Macroscopically and histologically, the OAB IV group exhibited a reduction in articular cartilage degeneration and less osteophyte formation and synovitis compared with the control group (no bevacizumab; OA group). Real-time PCR showed significantly lower expression of catabolic factors in the synovium in the OAB IV group compared with the OA group. In articular cartilage, expression levels of aggrecan, collagen type 2, and chondromodulin-1 were higher in the OAB IV group than in the OA group. Histological evaluation and assessment of pain behaviour showed a superior effect in the OAB IA group compared with the OAB IV group 12 weeks after administration of bevacizumab, even though the total dosage given to the OAB IA group was half that received by the OAB IV group.

**Conclusions:**

Considering the dosage and potential adverse effects of bevacizumab, the local administration of bevacizumab is a more advantageous approach than systemic administration. Our results suggest that intra-articular bevacizumab may offer a new therapeutic approach for patients with post-traumatic OA.

**Electronic supplementary material:**

The online version of this article (doi:10.1186/s13075-014-0427-y) contains supplementary material, which is available to authorized users.

## Introduction

Osteoarthritis (OA), the most common joint disease, is often given less attention than other diseases, such as cancer, because it is not a disorder directly associated with the sustainability of life. However, OA leads to severe joint dysfunction and pain, and a decline in the patient’s quality of life with an associated decrease in the ability to perform activities of daily life. Patients with early to mid-stage OA are given pharmacological treatment for pain relief, although the long-term benefits have not been shown convincingly. Patients with advanced OA are indicated for total joint arthroplasty.

Articular cartilage is an avascular tissue comprising a sparse cell population with low mitotic activity, and its capacity for self-repair is limited. Therefore, mature articular cartilage shows limited capacity for regeneration after degeneration or injury. For this reason, various treatments have been developed with the aim of restoring tissue quality via regenerative methods. Techniques such as microfracture [1], mosaicplasty [2], cell transplantation [3,4], and the implantation of tissue-engineered cartilage with [5-7] or without [8-10] various scaffolding materials have received increasing attention. However, the restorable areas are limited and tend to be replaced with bone or fibrocartilage tissue.

Previously, we investigated the use of an osteochondral defect model to explore methods to repair cartilage defect sites. This was first accomplished by developing a three-dimensional, scaffold-free, tissue-engineered cartilage [9] that was transplanted into osteochondral defects to initiate cartilage differentiation [10]. This method achieved good restorative effects in the long term, allowing us to confirm that articular cartilage repair can be achieved during the early stage of transplantation [10]. We noted that reparative cells from marrow had acquired anti-angiogenic properties, and we hypothesized that better cartilage repair might be achieved by inhibiting the bioactivity of vascular endothelial growth factor (VEGF) in osteochondral defects. We later reported that intravenous administration of an antibody against VEGF contributed to articular cartilage repair in an osteochondral defect model [11].

In OA, new blood vessels from the subchondral bone breach the tidemark into cartilage [12], and it is thought that these blood vessels contribute to articular cartilage ossification [13] and lead to osteophyte formation around the cartilage [14]. Angiogenesis and inflammation are closely integrated processes in the pathogenesis of OA, which is associated with increased angiogenesis in the synovium [15]. Synovitis is also characteristic of rheumatoid arthritis (RA). Studies of angiogenesis that have compared the pathogenesis of RA and OA have concluded that angiogenesis correlates with the extent of synovial hyperplasia observed in these two diseases and that hyperplasia is most severe in RA but is also present in OA-affected joints [16,17]. Angiogenesis also results in innervation of the articular cartilage [18], which may provide a source of pain in OA patients. Thus, an angiogenesis inhibitor that could suppress synovitis, osteophyte formation, and pain is an attractive candidate for the treatment of OA.

**Figure 1:**
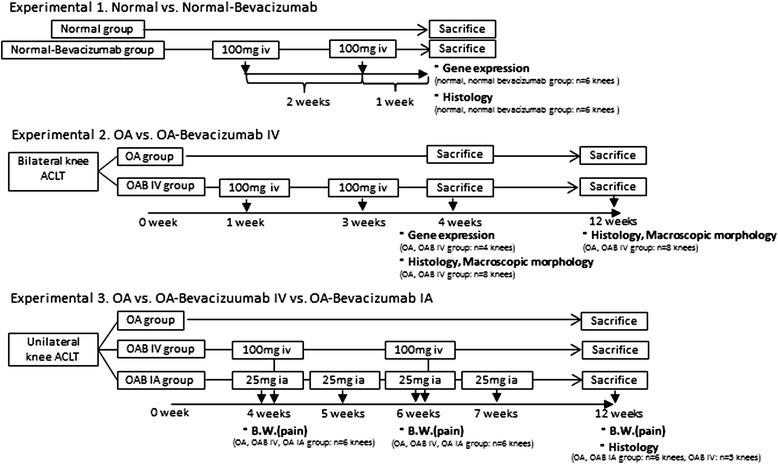
**Study outline.** This study comprised three experiments to assess the effects of bevacizumab in normal and rabbits with osteoarthritis (OA). Experiment 1: effects of bevacizumab treatment on normal articular cartilage, synovium, and subchondral bone were examined in 12 rabbits. Six normal (untreated) rabbits were given bevacizumab intravenously (iv) on day 1 and at 2 weeks (normal bevacizumab group, n = 12 knees), and the other six normal (untreated) rabbits were used as normal controls (normal group, n = 12 knees). All 12 rabbits were evaluated after 3 weeks to examine gene expression (normal bevacizumab group, n = 6 knees; normal group, n = 6 knees) and histology (normal bevacizumab group, n = 6 knees; normal group, n = 6 knees). Experiment 2: 20 rabbits were given bilateral anterior cruciate ligament transection (ACLT): 10 rabbits were given bevacizumab intravenously 1 week and 3 weeks after ACLT (OAB IV group), and 10 rabbits served as untreated OA controls (OA group). Rabbits were evaluated 4 weeks (n = 6 rabbits/group) and 12 weeks (n = 4 rabbits/group) after ACLT. Experiment 3: all rabbits were given unilateral knee ACLT. Intra-articular bevacizumab (25 mg) was injected 4 weeks after ACLT, then weekly until 7 weeks (OAB IA group). Intravenous bevacizumab (100 mg) was injected 4 and 6 weeks after ACLT (OAB IV group). Rabbits were evaluated 4, 6, and 12 weeks after ACLT. As the comparison, untreated unilateral ACLT rabbits were examined at the same times (OA group).

Although an anti-VEGF antibody is an attractive target for the treatment of neovascular disease, several complications associated with its intravenous administration have been reported, including haemorrhage, thromboembolism, proteinuria, delayed wound healing, and hypertension [19]. In a recent study, we showed that the systemic intravenous administration of bevacizumab improved articular cartilage repair in an osteochondral defect model [11]. In the current study, we first determined how normal joint tissue responds to bevacizumab treatment. We then evaluated the effects of intravenous injection of bevacizumab in a rabbit model of OA by comparing the restorative changes in the cartilage of the synovium, subchondral bone, and articular joint in bevacizumab-treated and untreated rabbits. Following this, we compared the effects on healing induced by intra-articular administration with those induced by intravenous administration of bevacizumab (Figure 1).

## Methods

### Animals and surgical procedures

Animal experiments were approved by the ethics review board of Tokai University and were performed in accordance with the guidelines on animal use of Tokai University (Authorization Number 122083). Adolescent Japanese white rabbits, aged 16 to 18 weeks and weighing about 2.5 kg, were used in this study. These rabbits were purchased from a professional breeder (Tokyo Jiken Dobutsu, Tokyo, Japan). The rabbits were anaesthetized by exposure to sevoflurane and O_2_ gas. Under sterile conditions, a medial parapatellar approach was used to release the joint capsule and to perform an anterior cruciate ligament transection (ACLT) [20]. After recovery from surgery, all animals were allowed to walk freely in their cages without any splints. Animals were sacrificed after the experiments by an overdose of intravenous anaesthesia.

### Bevacizumab dose

#### Intravenous (systemic) administration of bevacizumab

The half-life of bevacizumab in the normal blood circulation is reportedly 21.3 days [21]. In humans, the approved dose of bevacizumab is 5 mg/kg, with a clinical administration interval of more than 2 weeks [19]. Bevacizumab is cross-reactive with rabbit VEGF, but its affinity for rabbit VEGF is one-eighth that for human VEGF [22]. Therefore, we investigated bevacizumab at a dose of 40 mg/kg administered on day 1 and again 2 weeks later.

#### Intra-articular (local) administration of bevacizumab

The local administration of bevacizumab is currently performed in ophthalmology clinics, in which the drug is injected into the vitreous body at a concentration of 25 mg/ml [23]. Intravitreal bevacizumab has been shown to be non-toxic to the retina and optic nerve at the 2.5 mg dose tested in animals [24]. We thus chose to administer 25 mg/ml into the articular capsule of OA rabbits. A volume of 1 ml was deemed suitable because this is typical for intra-articular injections in rabbits [25].

### Experimental design

#### Experiment 1: normal versus normal bevacizumab

Twelve rabbits were used to investigate the effects of bevacizumab treatment on normal articular cartilage, synovium, and subchondral bone. Six rabbits were given 100 mg of bevacizumab intravenously on day 1 and then again 2 weeks later (normal bevacizumab group, n = 12 knees), and the other six rabbits were used as normal controls (normal group, n = 12 knees). All rabbits were sacrificed after 3 weeks to examine gene expression (normal bevacizumab group, n = 6 knees; normal group, n = 6 knees) and histology (normal bevacizumab group, n = 6 knees; normal group, *n* = 6 knees).

#### Experiment 2: OA versus OA intravenous bevacizumab

Twenty rabbits underwent bilateral ACLT. Ten rabbits were given an intravenous injection of 100 mg bevacizumab 1 week and 3 weeks after ACLT (OAB IV group). The other 10 rabbits were used as the untreated control group (OA group). It has been reported that, in unilateral and bilateral ACLT models, degenerative changes appear in the articular cartilage 4 weeks after surgery [26-28]. At 4 weeks, six rabbits from each group were sacrificed for histology (n = 8 knees) and gene expression (n = 4 knees). The remaining four rabbits in each group were sacrificed 12 weeks after ACLT, and their tissues were processed for histology (n = 8 knees).

#### Experiment 3: OA versus OA intravenous bevacizumab versus OA intra-articular bevacizumab

The final experiment was designed to compare intravenous with intra-articular administration of bevacizumab. In this experiment, ACLT was performed on 18 rabbits on only one knee per rabbit to measure pain behaviour in terms of weight-bearing asymmetry. Because ACLT was performed unilaterally in experiment 3, the progress of OA was predicted to be slower than that in the experiment involving bilateral ACLT. Therefore, we changed the timing of the administration of bevacizumab to 4 weeks after ACLT instead of 1 week. Six rabbits were given 100 mg of bevacizumab by intravenous injection 4 and 6 weeks after ACLT (OAB IV group, n = 6 knees, total dosage 200 mg), and six rabbits were given 25 mg bevacizumab by intra-articular injection 4, 5, 6, and 7 weeks after ACLT (OAB IA group, n = 6 knees, total dosage 100 mg). Six rabbits received no drug treatment (OA group, n = 6 knees). All animals were sacrificed 12 weeks after ACLT, and the knee joints were evaluated.

### Morphology of osteophyte formation

The femoral condyles were examined macroscopically. Morphological changes were evaluated by two independent observers. The criteria for macroscopic grading were as follows: grade 0 (absent), grade 1 (mild osteophyte formation), grade 2 (moderate osteophyte formation), and grade 3 (severe osteophyte formation), as described previously [29]. We included both osteophytes and chondro-osteophytes in our evaluation of osteophyte formation because both are regarded as neoplastic tissue caused by endochondral ossification as a result of angiogenesis in the articular margin associated with OA. The formation of chondro-osteophytes by hypertrophic chondrocytes reflects the process of endochondral ossification in the growth of osteophytes. The condyles were prepared for histological evaluation and gene expression analysis after morphological grading.

### Histological examination

The distal parts of the femur were excised and fixed in 4% paraformaldehyde for 7 days. Each specimen was decalcified in a solution of 10% ethylenediaminetetraacetic acid in distilled water (pH 7.4) for 2 to 3 weeks, embedded in paraffin wax, and cut along the sagittal plane. Each section was stained with Safranin O. We evaluated OA repair sites at the medial femoral condyle semiquantitatively using a grading and staging system (Osteoarthritis Research Society International (OARSI) modified Mankin score [30]). This system includes six histological grades and four histological stages. The total score (grade score multiplied by stage score) ranges from 1 point (normal articular cartilage) to 24 points (no repair). The sections were examined blindly by two observers, and the scores were averaged to minimize observer bias. The synovium was sampled from the infrapatellar fat pad region. The synovial membrane was fixed in 4% paraformaldehyde for 7 days and subsequently embedded in paraffin. Each section was stained with H&E or Masson trichrome stain.

### Gene analysis by real-time polymerase chain reaction (PCR)

The synovium was obtained from the infrapatellar fat pad region, articular cartilage was sampled from the femoral condyle, and subchondral bone was obtained from the femoral condyle with completely removed cartilage. Each construct was homogenized using a Cryo-Press (Microtec Nition, Chiba, Japan) in liquid nitrogen. Total RNA was isolated using the SV Total RNA Isolation System (Promega Corp., Madison, WI, USA), following the manufacturer’s instructions. RNA quantification and quality were determined using the 260/280 nm ratio. Each RNA sample was then reverse-transcribed to cDNA using TaqMan Reverse Transcription reagents (Applied Biosystems, Foster City, CA, USA) in a thermocycler set at 42°C for 60 minutes and at 95°C for 5 minutes. The primer sequences used in this study are listed in Additional file 1: Table S1. Real-time PCR was performed in an ABI SDS 7300 real-time PCR system (Applied Biosystems) using SYBR Green Master Mix (Applied Biosystems). cDNA (2 μl) was added to bring the final volume of the real-time PCR sample to 25 μl. We then ran 35 to 45 amplification cycles: 2 minutes at 50°C, 10 minutes at 95°C, 15 s at 95°C, 1 minute at 60°C, and then 15 s at 95°C, 30 s at 60°C, and 15 s at 95°C (dissociation step). Normal articular cartilage, synovium, or subchondral bone was used as the reference for comparisons of gene expression between samples in experiments 1 and 2. The relative expression of the target mRNA was standardized to glyceraldehyde phosphate dehydrogenase, and the expression level was calculated using the 2^−ΔΔCT^ values.

### Pain evaluation

An incapacitance test meter (Linton Instrumentation, Norfolk, England) was used to identify and compare trends in the weight distribution ratio of the undamaged versus damaged limbs. The measurements were obtained from rabbits after they were transferred into the rabbit holder. The weight distribution of both hind legs was measured 10 times, and the following formula was used to calculate the damaged limb weight distribution ratios (%) obtained by loading the left and right limbs:

$$ \begin{array}{l}\mathrm{Damaged}\ \mathrm{limb}\ \mathrm{weight}\ \mathrm{distribution}\ \mathrm{ratio}\kern0.5em \left(\%\right)=\hfill \\ {}\left\{\mathrm{Damaged}\ \mathrm{limb}\ \mathrm{load}\kern0.5em \left(\mathrm{g}\right)/\left(\mathrm{Undamaged}\ \mathrm{limb}\ \mathrm{load}\ \left(\mathrm{g}\right)+\mathrm{Damaged}\ \mathrm{limb}\ \mathrm{load}\ (g)\right)\right\}\times 100.\hfill \end{array} $$

### Statistical analysis

All data are expressed as the mean ± SD. The nonparametric Mann-Whitney *U*-test was used to compare gene expression, histology, and gross morphology between two groups (experiments 1 and 2). The Kruskal-Wallis test followed by post hoc comparisons (Mann-Whitney *U*-test) was used to compare the pain evaluation test results and histological data between three groups (experiment 3). Differences were considered significant when *P*-values were <0.05 for comparisons between two groups or <0.017 for comparisons between three groups.

## Results

### Experiment 1: Effect of bevacizumab in normal tissues

**Figure 2:**
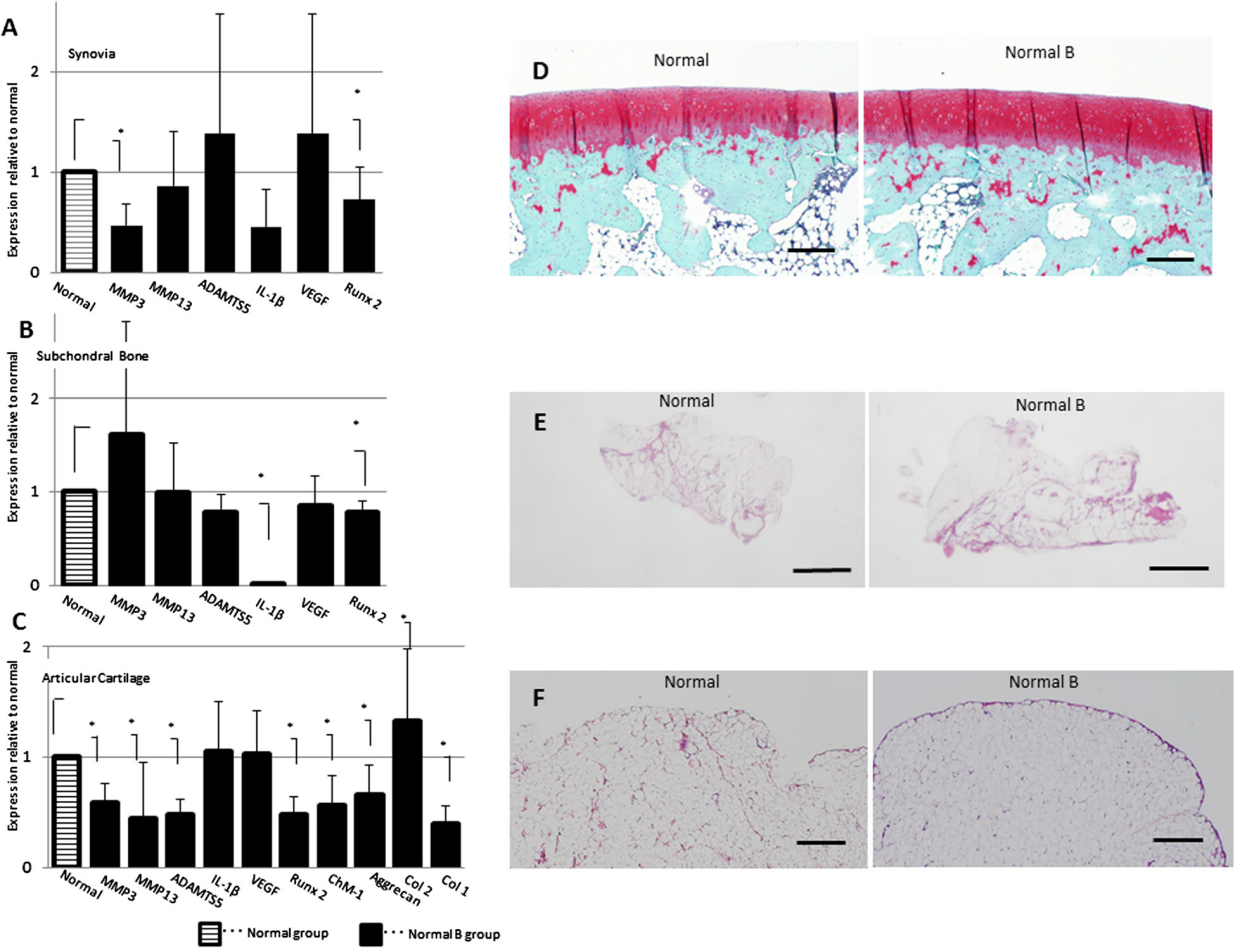
**Comparison of gene expression and histology between the normal bevacizumab and normal groups (experiment 1). (A)** Synovial gene expression. **(B)** Subchondral bone gene expression. **(C)** Articular cartilage gene expression. Gene expression data are expressed as mean ± SD (n = 6 knees). The y-axis is defined as follows: 2^–ΔCT^ normal B target gene/2^–ΔCT^ normal target gene. *P <0.05 by the Mann-Whitney *U*-test. **(D)** Representative articular cartilage histology for the normal group (left side) and normal bevacizumab group (right side) stained with Safranin O. **(E)** Representative synovial histology for the normal group (left side) and normal bevacizumab group (right side) stained with H&E. **(F)** Higher magnifications of the image in **(E)** stained with H&E. Histology data were obtained from six knees per group. Bars = 1 mm **(E)**; bars = 300 μm **(D, F)**.

Acute joint damage that occurs at the time of an injury initiates a sequence of events that can lead to progressive articular surface damage. Therefore, we sought to compare the gene expression and histological changes in the synovium, subchondral bone, and articular cartilage of knees between normal untreated (normal group) and bevacizumab-treated normal rabbits (normal bevacizumab group) (Figure 2). We administered bevacizumab intravenously and examined changes in the gene expression levels of various catabolic factors, including matrix metalloproteinase-3 (MMP3), MMP13, ADAMTS5 (a disintegrin and metalloproteinase with thrombospondin motifs-5), and interleukin-1β (IL-1β). In the synovium, the expression of most genes tended to be lower in the normal bevacizumab group compared with the normal group; *MMP3* and *Runx2* expression levels were significantly lower in the normal bevacizumab group (Figure 2A). In subchondral bone, *IL1B* and *Runx2* expression levels were significantly lower in the normal bevacizumab group than in the normal group (Figure 2B). In articular cartilage, the expression levels of *MMP3*, *MMP13*, and *ADAMTS5* were significantly lower in the normal bevacizumab group compared with the normal group (Figure 2C). In the articular cartilage, the expression level of collagen type 2, the major collagen in cartilage, was elevated, but that of collagen type 1, the major collagen in bone and tendon, was lower in the normal bevacizumab group compared with the normal group. The expression levels of chondromodulin-1 (*ChM-1*), an endogenous anti-angiogenic factor, and aggrecan, the major structural protein in cartilage, were also lower in the normal bevacizumab group compared with the normal group. Intriguingly, *VEGF* mRNA expression was unaffected by intravenous administration of bevacizumab (Figure 2A-C).

Joints from the normal bevacizumab group showed smooth and uniform articular surfaces and Safranin O staining throughout the articular cartilage, similar to the appearance in the normal group (Figure 2D). In the synovium in the normal bevacizumab group, there was no synoviocyte hyperplasia or proliferation, or lymphoplasmacytic infiltration. One layer of synovial membrane with underlying adipose cells was apparent (Figure 2E, F).

### Experiment 2: Comparison between OA and OA intravenous bevacizumab

We next studied the effects of bevacizumab in a rabbit model of OA, in which OA was induced by ACLT. Bevacizumab was administered 1 and 3 weeks after induction of OA into 10 rabbits (OAB IV group); 10 untreated OA-only rabbits served as controls (OA group). All rabbits were assessed for morphological differences and histological changes at 4 and 12 weeks, and gene expression changes were assessed at 4 weeks.

#### Morphology of osteophyte formation

**Figure 3:**
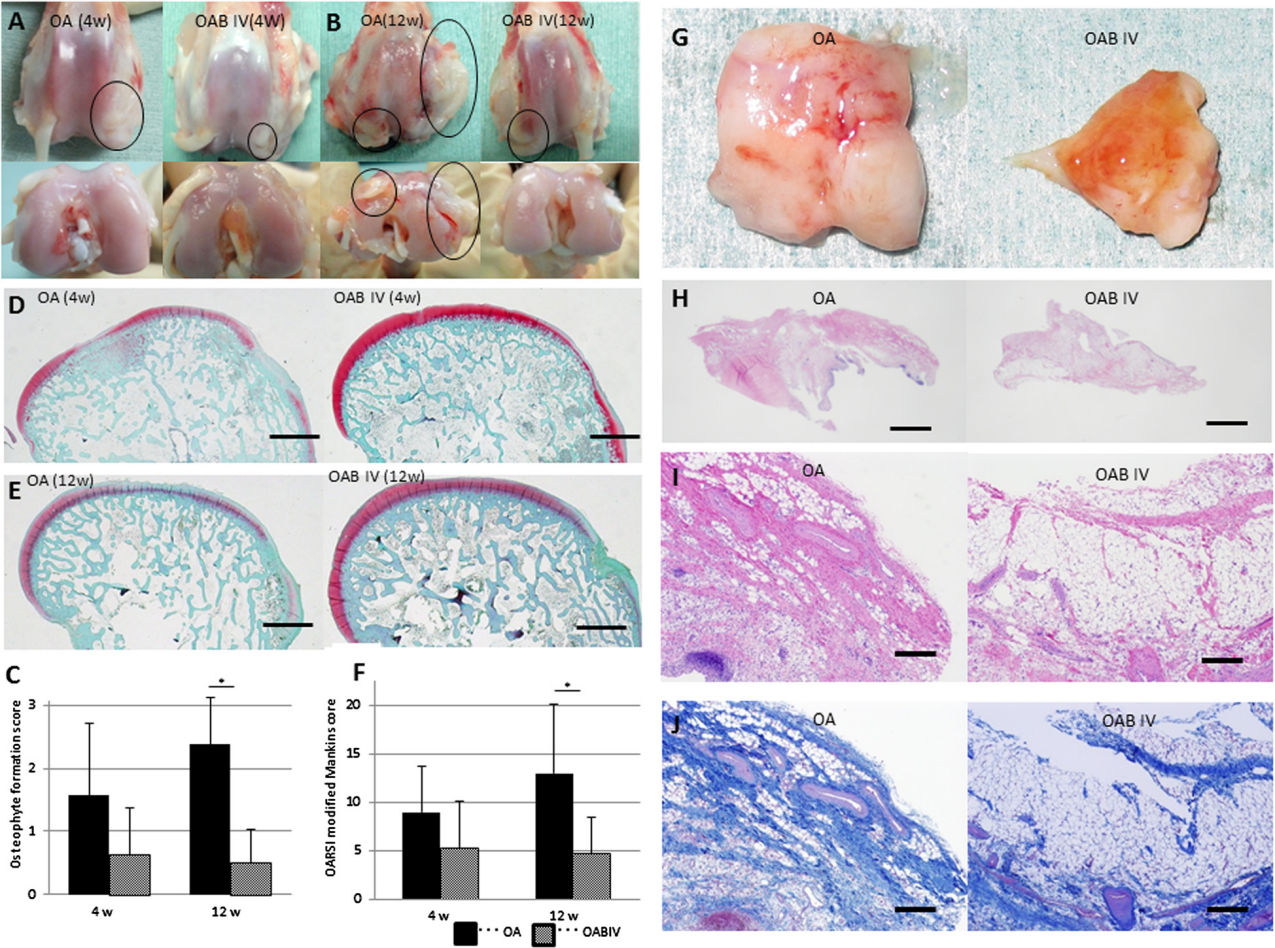
**Comparison of macroscopic and histological evaluations between the osteoarthritis (OA) and intravenous administration of bevacizumab in OA (OAB IV) rabbits (experiment 2). (A)** Macroscopic articular cartilage appearance 4 weeks after anterior cruciate ligament transection (ACLT). **(B)** Macroscopic appearance 12 weeks after ACLT. Circles indicate osteophyte formation. **(C)** Osteophyte formation scores 4 and 12 weeks after ACLT. Data are expressed as mean ± SD (n = 8 knees). Safranin O staining of hyaline cartilage 4 weeks **(D)** and 12 weeks **(E)** after ACLT. **(F)** Osteoarthritis Research Society International histological score 4 and 12 weeks after ACLT. **(G)** Macroscopic synovia appearance 4 weeks after ACLT. **(H)** Representative synovial histology for OA group (left side) and OAB IV group (right side) stained with H&E. Higher magnifications of the image shown in **(H)** stained with HE **(I)** and Masson trichrome **(J)**. Data are expressed as mean ± SD (n = 8 knees). *P <0.05 by the Mann-Whitney *U*-test. Bars = 1 mm **(D, E, H)**; bars = 300 μm **(I, J)**.

Four weeks after ACLT, macroscopic evaluation of joints from both groups identified osteophyte formation but nearly completely smooth joint surfaces of the articular cartilage (Figure 3A). The osteophyte formation score did not differ between the OA group and the OAB IV group (Figure 3C). Twelve weeks after ACLT, the joint surfaces showed a marked progression of arthritis and osteophyte formation in the OA group. The joints from the OAB IV group retained smooth joint surfaces in most regions of the articular cartilage and had significantly less osteophyte formation (Figure 3B, C).

#### Histological evaluation of the articular cartilage

We evaluated the medial femoral condyle at the femoral-tibial (FT) site in the articular cartilage area. Four weeks after ACLT, histological assessment showed a loss of Safranin O-positive staining in the OA group, whereas staining was retained in the OAB IV group (Figure 3D). By 12 weeks after ACLT, the joints in the OA group showed delamination of the superficial layer, erosion of hyaline cartilage, and a lack of Safranin O staining. Joints from the OAB IV group showed smooth and uniform articular surfaces and Safranin O staining throughout the articular cartilage (Figure 3E). The total OARSI histological score did not differ between the OA and OAB IV groups at 4 weeks but was significantly lower in the OAB IV group than in the OA group at 12 weeks (Figure 3F).

#### Morphology and histology of the synovium

Four weeks after ACLT, the macroscopic appearance of the synovium showed enlargement with redness and recognized thick vascular invasion in the OA group. By contrast, in the OAB IV group, the synovium was preserved and appeared pale yellow, although a thin vascular invasion was apparent (Figure 3G). Synovial sections from the OA group showed severe synoviocyte hyperplasia with diffuse lymphoplasmacytic infiltration, blood vessel proliferation, and severe fibroblast proliferation with regression of adipose cells. These characteristics were present in the OAB IV group, but they were more moderate (Figure 3H, I, J). These results indicate that the structural damage was less severe in the OAB IV group than in the OA group.

#### Gene expression

**Figure 4:**
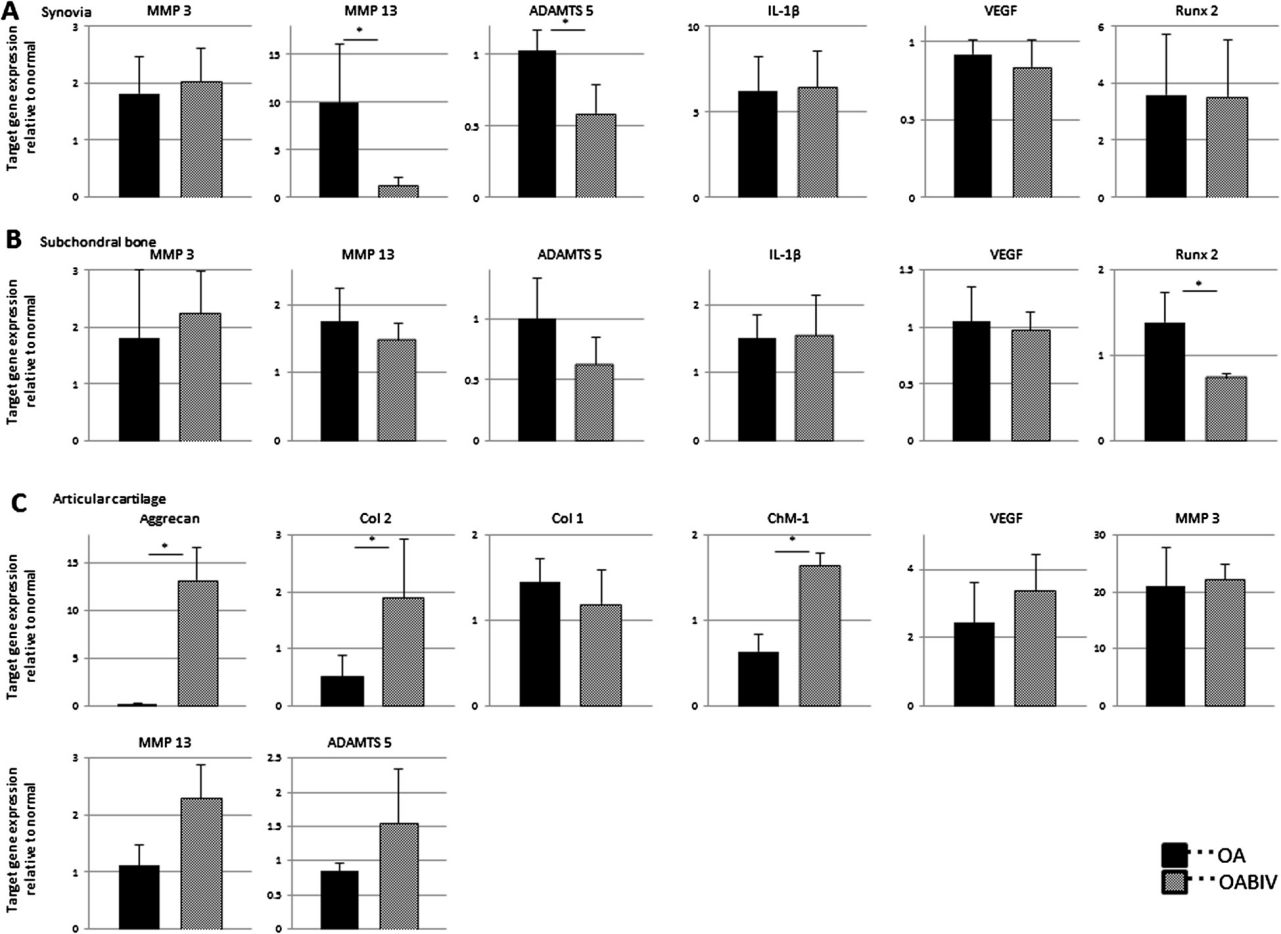
**Comparison of relative gene expression levels between the osteoarthritis (OA) and intravenous administration of bevacizumab in OA (OAB IV) groups (experiment 2). (A)** Synovial gene expression. **(B)** Subchondral bone gene expression. **(C)** Articular cartilage gene expression. The y-axis is defined as follows: 2^–ΔCT^ OA or OAB IV target gene/2^–ΔCT^ normal target gene. The normal tissue is represented as 1 on the y-axis. Data are expressed as mean ± SD (n = 4 knees). *P <0.05 by the Mann-Whitney *U*-test.

We harvested the synovium, subchondral bone, and articular cartilage from the OA and the OAB IV groups 4 weeks after ACLT. We used real time PCR to assess changes in the expression of genes involved in anabolic and catabolic factors, and we compared these changes between the OA and OAB IV groups relative to the expression levels of the normal tissues at the baseline. *MMP13* gene expression in the normal synovium was about 10-fold higher in the OA group than in untreated animals. *MMP13* gene expression in the OAB IV group was one-tenth that in the OA group, a level that approximated the normal level. *ADAMTS5* gene expression did not increase in the OA group compared with the normal tissue but was significantly decreased by administration of bevacizumab (Figure 4A). The effect of bevacizumab on the OA synovial tissue differed from that in the normal synovium (Figure 2A). In subchondral bone, *Runx2* expression was also significantly lower in the OAB IV group compared with the OA group (Figure 4B). However, bevacizumab had a similar inhibitory effect on *Runx2* expression as in normal subchondral bone (Figure 2B).

Articular cartilage from the OAB IV group showed significantly 3.5-fold higher collagen type 2 gene expression compared with the OA group, and there was a tendency toward lower collagen type 1 gene expression in the OAB IV group compared with the OA group (Figure 4C). The patterns of changes in the expression of types 1 and 2 collagen genes were similar to those induced by bevacizumab treatment in normal articular cartilage (Figure 2C). Unlike the effect of bevacizumab in normal tissue, aggrecan and *ChM-1* gene expression levels were significantly higher in the OAB IV group compared with the OA group. By contrast, *MMP13* and *ADAMTS5* gene expression levels were about 2.0-fold higher and *VEGF* gene expression level 1.4-fold higher in the OAB IV group than in the OA group, but this increase was not significant. *MMP3* gene expression levels did not differ between the OAB IV group and the OA group.

### Experiment 3: differences between intravenous and intra-articular administration of bevacizumab in a model of OA

#### Damaged limb weight distribution ratio (%)

**Figure 5:**
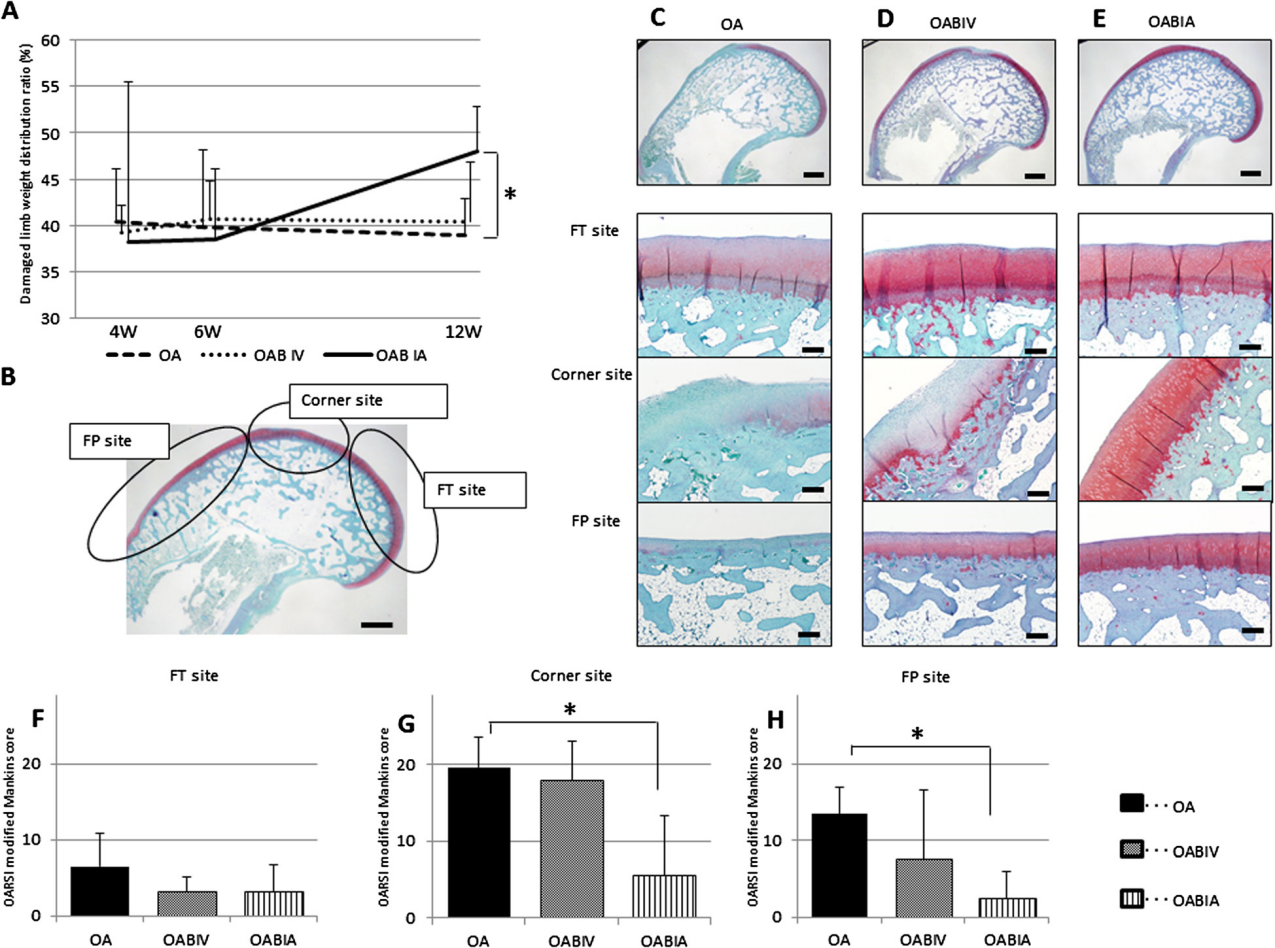
**Comparison of pain and histological evaluations between osteoarthritis (OA), intravenous administration of bevacizumab in OA (OAB IV), and intra-articular administration of bevacizumab in OA (OAB IA) groups (experiment 3). (A)** An incapacitance test meter was used to investigate the pain-ameliorating effects of treatment. The damaged limb weight distribution ratio (%) = {Damaged limb load (g)/(Undamaged limb load (g) + Damaged limb load (g))} × 100. The damaged limb weight distribution ratios 4, 6, and 12 weeks after surgery are shown. Data are expressed as mean ± SD (4 and 6 weeks after surgery: for each group, n = 6 knees; 12 weeks after surgery: OA and OAB IA groups, n = 6 knees, OAB IV group, n = 5 knees). *P <0.05 (Kruskal-Wallis post hoc test and Mann-Whitney *U*-test). **(B)** Distribution of femoral-patellar (FP), corner, and femoral-tibial (FT) sites. Safranin O-stained sections of articular cartilage. Bar = 1 mm. Representative Safranin-O-stained sections for the **(C)** OA, **(D)** OAB IV, and **(E)** OAB IA groups. Top row, overview of the distal femur. Bar = 1 mm. Second, third, and fourth rows, enlarged images of the FT site, corner site, and FP site. Bar = 250 μm. Osteoarthritis Research Society International histological scoring was performed at the **(F)** FT, **(G)** corner, and **(H)** FP sites. Data are mean ± SD (OA and OAB IA groups: n = 6 knees; OAB IV group: n = 5 knees). *P <0.05 (Kruskal-Wallis post hoc test and Mann-Whitney *U*-test).

A comparison of the weight distribution ratio between the three groups (OA, OAB IV, and OAB IA) following bevacizumab treatment is shown in Figure 5A. Up to 6 weeks, the rabbit weight distribution ratio did not differ between the three groups. However, by 12 weeks, rabbits in the OAB IA group showed a satisfactory improvement in the damaged limb weight distribution ratio, whereas rabbits in the OA and OAB IV groups failed to show such improvement. The OAB IA group showed significant improvement compared with the OA group (Figure 5A). Unfortunately, one rabbit from the OAB IV group developed diarrhoea and lost weight, and this rabbit died from complications involving digestion at 8 weeks.

#### Histological evaluation

Because unilateral ACLT was performed, the OA change was not as progressive at the FT site compared with that observed in rabbits that had received the bilateral ACLT in experiment 2. Therefore, we divided the distal portion of the femur into the FT site, femoral-patellar (FP) site, and corner site, which was between FP and FT (Figure 5B). In the OA group, diminished Safranin O staining and clear degeneration of the cartilage were observed at the FT site. Only minimal Safranin O staining was observed at the corner and FP sites (Figure 5C). By contrast, in the OAB IV group, matrix staining was strong at the FT site throughout the thickness of the cartilage and was stronger at the corner and FP sites compared with the OA group. Some matrix staining depletion was observed within the upper third of the cartilage at the corner site and within the full thickness of the cartilage within the FP site (Figure 5D). In the OAB IA group, matrix staining depletion was minimal at all three sites, and staining was observed throughout the full thickness of the tissue (Figure 5E). As expected, the OARSI histological score for Safranin O staining at the FT site did not differ significantly between the three groups (Figure 5F). However, at the corner and FP sites, the histological score was significantly lower in the OAB IA group than in the OA group (Figure 5G, H). These results indicate that the OAB IA group had less OA progression compared with the OAB IV and OA groups.

## Discussion

We previously demonstrated that the intravenous administration of bevacizumab repaired articular cartilage in an osteochondral defect model [11]. Based on those results, the present study was designed as three separate experiments to (1) assess the effect of bevacizumab in normal joints; (2) clarify the therapeutic efficacy of the intravenous administration of bevacizumab in OA joints; and (3) evaluate the effects of a systemic versus local administration of bevacizumab in OA joints.

The intravenous administration of 100 mg of bevacizumab for a period of 2 weeks did not lead to inflammation or angiogenic effects in the three tissue types in the normal joint as determined by histological and gene expression in experiment 1. The expression of the genes for some anabolic and catabolic factors was suppressed by administration of bevacizumab in normal articular cartilage. This environment may be the extremely stable hypo-metabolism, rather than the toxicity to cartilage with bevacizumab.

Anderson *et al.* recently reported that early molecular interventions can limit cartilage degradation by minimizing the extent of tissue damage and can help accelerate healing by decreasing the inflammatory response [31]. In the current study, early intervention by intravenous administration of bevacizumab appeared to contribute to a reduction in articular cartilage degeneration, osteophyte formation, and synovitis, as determined macroscopically and histologically in experiment 2.

We performed PCR analyses to assess whether bevacizumab has preventive effects on the whole joint (that is, by comparing the results of experiments 1 and 2). In the synovium, *MMP13* and *ADAMTS5* expression levels were significantly lower in the OAB IV group than in the OA group. Although bevacizumab did not decrease *IL1B* expression significantly, we suggest that the decrease in *MMP13* and *ADAMTS5* expression levels contributed to the absence of synovitis.

Higher VEGF mRNA and protein levels have been reported in the synovial tissue of patients with inflammatory joint disease who required synovectomy compared with healthy control subjects [32]. Acute joint damage that occurs at the time of an injury initiates a sequence of events that can lead to progressive articular surface damage [31]. Subsequently, fragments of cartilage can induce synovitis in these patients [33]. In this study, damage to the articular surface led to an elevation in *VEGF* expression in the articular cartilage. In the synovium, there was a strong increase in the expression of *MMP* and *IL1B*, which indicates inflammation, but *VEGF* expression, which represents angiogenesis, was unchanged. This is probably because our study examined the early stages of OA (4 weeks after ACLT). Therefore, it seems likely that *VEGF* expression in the synovial tissue was not influenced by bevacizumab treatment.

*Runx2* is the master gene of bone formation and contributes to sclerosis in subchondral bone and the formation of osteophytes [34]. From the early stages in our model, we observed a significant decrease in *Runx2* expression with bevacizumab treatment, suggesting that bevacizumab contributes to the inhibition of osteosclerosis in the subchondral bone and inhibits osteophyte formation.

In this study, the expression of both *VEGF* and *ChM-1* was examined in the articular cartilage. We consider that the balance between angiogenic and anti-angiogenic factors is important to articular chondrocyte survival and preservation of the phenotype. The expression of *ChM-1*, which was lower in the OA group than in the normal group, increased with bevacizumab treatment. ChM-1 is reported to be a strong inhibitor of angiogenesis involved in the development of bone and regulation of vascular invasion during endochondral bone formation [35]. Articular cartilage contains considerable amounts of ChM-1, which should induce the chondrocyte phenotype [36]. It has been posited that the development of the OA phenotype might result from articular chondrocytes undergoing a differentiation route reminiscent of growth plate chondrocytes and expressing hypertrophy-like changes [37]. Thus, ChM-1 may act to prevent chondrocyte hypertrophy and to stabilize the articular chondrocyte phenotype [38].

VEGF is necessary for chondrocyte survival during cartilage development [39]. In an osteochondral defect model, we showed previously that administration of bevacizumab caused ChM-1 to accumulate in the interterritorial space of the repaired matrix surrounding the chondrocytes, which expressed VEGF [11]. In the articular cartilage, although *VEGF* expression was 2.4-fold higher in the OA group than in normal tissue from untreated rabbits, bevacizumab did not significantly alter *VEGF* expression.

The gene expression levels of the anabolic factors aggrecan and collagen type 2 were significantly higher in the OAB IV group than in the OA group. Interestingly, the gene expression of the catabolic factors in the articular cartilage, MMP13 and ADAMTS5, were higher in the OAB IV group than in the OA group, although this difference was not significant. Because this study was performed *in vivo*, we consider that the expression of the catabolic genes may have led to degradation of the existing matrix and subsequent proliferation and synthesis of new matrix in the limited articular cartilage space. The increase in *ChM-1* expression in response to bevacizumab may have increased the anabolic and catabolic factors, leading to proliferation and synthesis of new matrix *in vivo*. Interestingly, however, in normal articular cartilage, *ChM-1* expression was decreased by administration of bevacizumab (Figure 2C). Kitahara *et al*. suggested that ChM-1 acts to inhibit vascular invasion in the immature state of articular cartilage and that ChM-1 levels decrease gradually with age thereafter [40]. Such findings suggest a regulatory role of ChM-1 in vascular invasion; that is, exposure of tissues to angiogenesis factors is reduced in mature articular cartilage concomitant with a reduction in ChM-1 at both the protein and gene expression levels. In other words, ChM-1 appears to decrease at the non-angiogenic sites. In the current study, it is possible that angiogenic factors were reduced at the protein level after bevacizumab administration in the normal articular cartilage; if so, *ChM-1* gene expression would have also decreased following the administration of bevacizumab in normal articular cartilage.

One limitation of this study is that we did not measure VEGF protein levels in the tissue before and after treatment with bevacizumab, which is important when examining the treatment effects of bevacizumab. In this experiment, intermittent treatment with bevacizumab induced less OA progression, as shown by both histological and gene expression assays, through increases in the gene expression of *ChM-1* and *VEGF* in the articular cartilage and decreases in the gene expression of *MMP 13* and *ADAMTS5* in the synovium. Thus, although bevacizumab bound to VEGF protein, this did not prevent the expression of *VEGF*, which is needed for cartilage survival. In addition, it is thought that bevacizumab functions to modify the local environment by preventing angiogenesis in articular cartilage and synovial inflammation. Further research is required to understand better what determines the magnitude of the increase in ChM-1 at both the protein and gene expression levels in response to bevacizumab in OA tissues. It is important to understand the pharmacokinetic profile of the drug if we are to optimize dosing regimens for patients and to obtain a better assessment of its safety profile.

The second limitation of this study is that we did not monitor the dose-response relationship for bevacizumab because a sufficient preventive effect against OA progression occurred with the intravenous administration of 200 mg (2 × 100 mg) of bevacizumab and intra-articular administration of 100 mg (4 × 25 mg) of bevacizumab. In the absence of intravenous administration of bevacizumab, intra-articular administration may lead to bevacizumab being absorbed by the highly vascular synovial membrane and then circulated throughout the whole body. Therefore, some of the effects of the intra-articular injection of bevacizumab may be attributable to its presence in the systemic circulation. Future studies should assess the effects of tapered dosages of bevacizumab and the optimal systemic dosage.

We have shown here that intravenous administration of bevacizumab is associated with reductions in synovitis, articular degeneration, and osteophyte formation in an ACLT model of OA. Systemic bevacizumab-treated joints also generally showed greater aggrecan preservation, as indicated by the intensity of Safranin O staining of the articular cartilage (Figure 3). These results are promising for translating this study into the clinical setting. As described earlier, there are numerous potential side effects of systemic administration of bevacizumab. It is likely that the intra-articular administration of bevacizumab will decrease the risk of these adverse events. In this study, there was no disadvantage associated with intra-articular administration of bevacizumab compared with intravenous administration. The total dose of the drug given to the OAB IA group was half that given to the OAB IV group. Moreover, the animals exhibited significantly less pain in the OAB IA group. Bonnet and Walsh commented that angiogenesis may introduce sensory nerves into the aneural cartilage and that inflammation can sensitize nerves present in the joint [33]. It is believed that the intra-articular administration of bevacizumab inhibits angiogenesis and inflammation, thereby reducing OA progression and providing pain relief. Because ACLT was performed unilaterally in experiment 3, the progression of OA was slower than that in experiment 2, which involved bilateral ACLT. Similar to experiment 2, the differences in the histological score and pain behaviour between the OA and OAB IV groups in experiment 3 will seem to occur after 12 weeks. Intravenous administration of bevacizumab is a promising treatment option for polyarthritis, whereas intra-articular administration might be recommended for patients with single-site arthritis.

## Conclusions

In conclusion, we show that bevacizumab reduced synovitis, osteophyte formation, and cartilage degradation in a rabbit model of OA. Importantly, intra-articular administration of bevacizumab also reduced pain behaviour. Because bevacizumab was administered before the development of structurally severe OA, its effects might reflect changes in synovitis rather than direct effects on cartilage pathogenesis. Our findings indicate that early intervention by the administration of bevacizumab following an ACL injury may be beneficial in retarding the development of post-traumatic OA.

## Additional file

Additional file 1
**List of primers used in real-time PCR.**

## Abbreviations

ACLT: anterior cruciate ligament transection
ADAMTS5: A disintegrin and metalloproteinase with thrombospondin type 1 motif-5
ChM-1: chondromodulin-1
ACL: anterior cruciate ligament
FP: femoral-patellar
FT: femoral-tibia
H&E: haematoxylin and eosin
IL-1β: interleukin-1β
MMP: matrix metalloproteinase
OA: osteoarthritis
OAB IA: intra-articular administration of bevacizumab in OA
OAB IV: intravenous administration of bevacizumab in OA
OARSI: Osteoarthritis Research Society International
PCR: polymerase chain reaction
RA: rheumatoid arthritis
Runx2: runt-related transcription factor-2
VEGF: vascular endothelial growth factor
