# Supplementary figures and images for: Bevacizumab, an anti-vascular endothelial growth factor antibody, inhibits osteoarthritis
Source: Arthritis Res Ther. 2014 Sep 18;16(5):427. doi: 10.1186/s13075-014-0427-y (PMC4189677; doi:10.1186/s13075-014-0427-y)

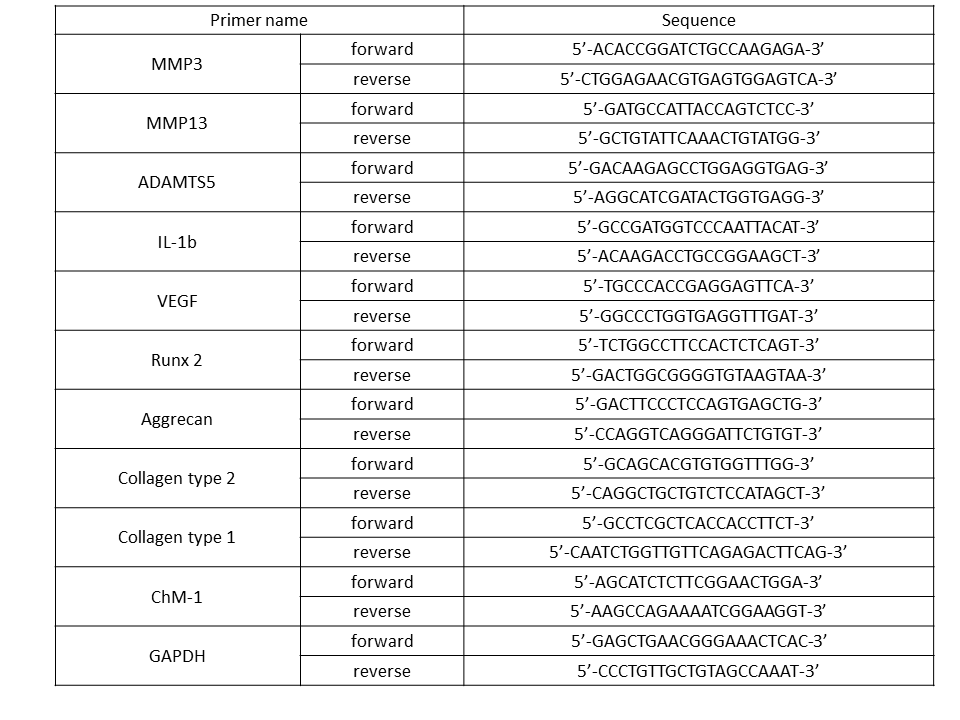

Supplement: Additional file 1 — List of primers used in real-time PCR. [file 13075_2014_427_MOESM1_ESM.tiff]
